# Backward and forward neck tilt affects perceptual bias when interpreting ambiguous figures

**DOI:** 10.1038/s41598-022-10985-4

**Published:** 2022-05-04

**Authors:** Fumiaki Sato, Ryoya Shiomoto, Shigeki Nakauchi, Tetsuto Minami

**Affiliations:** Department of Computer Science and Engineering, Electronics-Inspired Interdisciplinary Research Institute (EIIRIS), Toyohashi University of Technology, 1-1 Hibarigaoka Tempaku, Toyohashi, Aichi 441-8580 Japan

**Keywords:** Neuroscience, Physiology, Psychology

## Abstract

The relationships between posture and perception have already been investigated in several studies. However, it is still unclear how perceptual bias and experiential contexts of human perception affect observers’ perception when posture is changed. In this study, we hypothesized that a change in the perceptual probability caused by perceptual bias also depends on posture. In order to verify this hypothesis, we used the Necker cube with two types of appearance, from above and below, although the input is constant, and investigated the change of the probability of perceptual content. Specifically, we asked observers their perception of the appearance of the Necker cube placed at any of the five angles in the space of virtual reality. There were two patterns of neck movement, vertical and horizontal. During the experiment, pupil diameter, one of the cognitive indices, was also measured. Results showed that during the condition of looking down vertically, the probability of the viewing-from-above perception of the Necker cube was significantly greater than during the condition of looking up. Interestingly, the pupillary results were also consistent with the probability of the perception. These results indicate that perception was modulated by the posture of the neck and suggest that neck posture is incorporated into ecological constraints.

## Introduction

Human vision is flexible, and when individuals encounter an ambiguous object that can be perceived in multiple ways, the image of the object is processed according to practical rules such as those obtained through learning, known as “heuristics”. Numerous researchers have proposed visual heuristics for 2D estimation, such as light source estimation (when convex–concave ambiguity occurs, it is assumed the light source is above the object [strictly, it may be biased to the left or right instead of directly above])^1^; the generic view principle (the visual system works as if it were viewing from a general viewpoint rather than accidental viewpoints)^2^; and finally, the viewing-from-above bias (when ambiguous figures such as a Necker cube are observed, the observer tends to choose a viewpoint from above rather than from below)^3^ (see also a review^4^).

Although many studies have suggested the effect of perceptual heuristics, most have investigated this concept by presenting the stimulus in front. Specifically, observers in many experiments encountered the stimulus by sitting on a seat and looking directly in front of them. Therefore, the relationship between posture and perceptual heuristics is unclear, and it remains unknown if the heuristics that accompany physical changes (i.e., posture changes) affect perception.

Classically, Gibson, who advocated for ecological psychology, recommended considering perception both for the stimuli and the environment^5^. If the general view is implicitly defined in the observer, as in the idea of the generic view principle, the perceived experience and its principle when looking up and down should be different. For example, in our daily life, it is easy to perceive the sun and light sources when facing up and the ground when facing down. Previous studies suggest that recognizing the ground affects the perception of the size of objects^6^. Thus, such different upper and lower perceptual experiences were assumed to be associated with posture changes.

The relationship between posture and perception has been investigated in several studies; these have shown that size^7^, apparent size, and brightness^8^ vary depending on the position (or orientation) of the head. These phenomena are explained by proprioceptive theory, which proposes that the size and brightness of an object are learned in a natural environment; thus, an abnormal posture causes misestimation. In addition, studies have also investigated the relationship between head orientation and depth estimation^9,10^. The physiological basis of this hypothesis is that proprioceptive information from the somatosensory area in the postcentral gyrus is integrated with visual information^11,12^.

In this study, we extended this theory and investigated whether slight changes in posture, which occur in daily life, also cause perceptual changes. Specifically, we investigated whether the perceptual content changes due to modifications in the posture of the neck. To investigate this effect, we used the Necker cube, which is an ambiguous figure. The Necker cube can be perceived as having two appearances—one as a cube seen from above and the other from below. Further, the perceived probability of the changes in appearance depends on priming^13^, top-down intention^14^, eye movement^15^, and eye position^16^. Moreover, the probability of initial perception has been shown to be greater when an object’s appearance is perceived from above rather than from below and reflects the VFA bias^17^. Taken together, perceived probability changes due to various factors. In this study, we hypothesized that such changes in probability caused by perceptual bias are also dependent on posture.

In a previous related study, participants’ behaviors were shown to affect perceptual bias^18^. This study used a stimulus constituting a structure-from-motion cylinder, which may be perceived as rotating either clockwise or counterclockwise, to investigate how participants’ behavior affected perception. The results suggested that perception was formed by linking visual input and motor function, which may be related to the sensorimotor system. However, in this experiment, the action input occurred concurrently with the visual input. Therefore, the following question remains: is perception modulated not only by “action” but also by “posture”? This question is important in elucidating how humans form visual perception.

We devised a paradigm that presents the 2D Necker cube in 3D space using a head-mounted display (HMD) to answer this question. The setting constituted the participant’s face looking front at 0 degrees, and we measured perceptual probability and pupil diameter when facing five different angles (-60, -30, 0, 30, and 60 degrees), vertically and horizontally. To ensure test rigor, we also set the horizontal condition. This condition represents the “non-experiential” posture perception context and should not affect Necker cube perception, in contrast to the vertical condition, which constitutes the “experiential” posture perception context.

Pupil diameter reflects cognitive factors such as attention^19,20^, memory^21,22^, cognitive load^23^, visual context^24^, and top-down effect (see reviews^25–27^). The top-down effect as reflected by pupil diameter represents a decision-making process based on attention and is influenced by the observer’s experience, learning, and cognitive state. Typically, when viewing an image of the sun, pupil diameter decreases despite brightness being controlled^28^. It has also been shown that pupil diameter changes according to the size of action-congruency effects in a visual action-planning task^29^. Action-congruency effects refer to behavior consistent with a stimulus, which improves perceptual performance. Pupillometry can be used to estimate perceptual state when viewing the Necker cube^30^. Therefore, we used the pupil index to test whether the changes in perceptual probability accompanying changes in posture could be tracked.

To empirically examine the experiential context effect on perception according to neck posture, in addition to the vertical condition, we incorporated the horizontal condition, which corresponds to the non-experiential postural position. In addition, we applied the cueing paradigm to test whether prior information affected perception and differed according to neck posture^30^. The presentation of the priming cue of an unambiguous cube for a short time was observed to subsequently stabilize the appearance of the Necker cube^30,31^. Based on this evidence, we assumed a stable behavioral response and pupillary response could be obtained without perceptual switching. Combining these techniques, this study aimed to clarify the relationship between neck posture and visual heuristics from the aspects of both behavioral response and pupil diameter, an established physiological cognitive index.

## Materials and methods

### Participants

Twenty-five healthy individuals participated in Experiment 1 (mean age = 21.84 years, SD = 1.03; 24 men, one woman). One participant who could not provide data due to mechanical problems and one participant who misunderstood the instruction of the behavioral task were excluded from the data analysis of Experiment 1; thus, 23 responses were obtained for the final analytic sample. In Experiment 2, 19 healthy individuals participated (mean age = 22.3 years, SD = 1.05; 19 men). To estimate the necessary sample size, a priori power analysis was performed using PANGEA (Power ANalysis for GEneral ANOVA designs; see also https://www.jakewestfall.org/pangea)^32^. Assuming a medium effect size (effect size (*d)* = 0.45), *α* = 0.05 and β (statistical power) = 0.95 were set. In the design of Experiment 1, we were interested in both three-way and two-way interactions; hence, we calculated the number of participants with statistical powers above 0.95 in both cases. In the design of Experiment 2, the number of participants was computed fixed to a second-order interaction. According to the calculations, the number of participants was estimated to be 22 in Experiment 1 and 23 in Experiment 2. These sample sizes are considered reasonable because they were similar to those in a previous study investigating perceptual bias of a Necker cube using pupillometry^30^. We recruited students from the university based on the estimated sample size. In Experiment 2, the number of applicants did not reach the target sample size; therefore, post hoc analysis was performed. The statistical power was 0.99 based on the effect size of the result of Experiment 2 and the number of participants. In both experiments, the male–female ratio of the subjects was biased, but since gender would not play a role in the illusory effect and/or pupil response, we did not focus on equal representation of gender/men or women. The experimental procedures were approved by the Committee for Human Research at Toyohashi University of Technology. Participants provided written informed consent, and the experiment was conducted in accordance with the guidelines of the committee.

### Stimuli and apparatus

**Figure 1:**
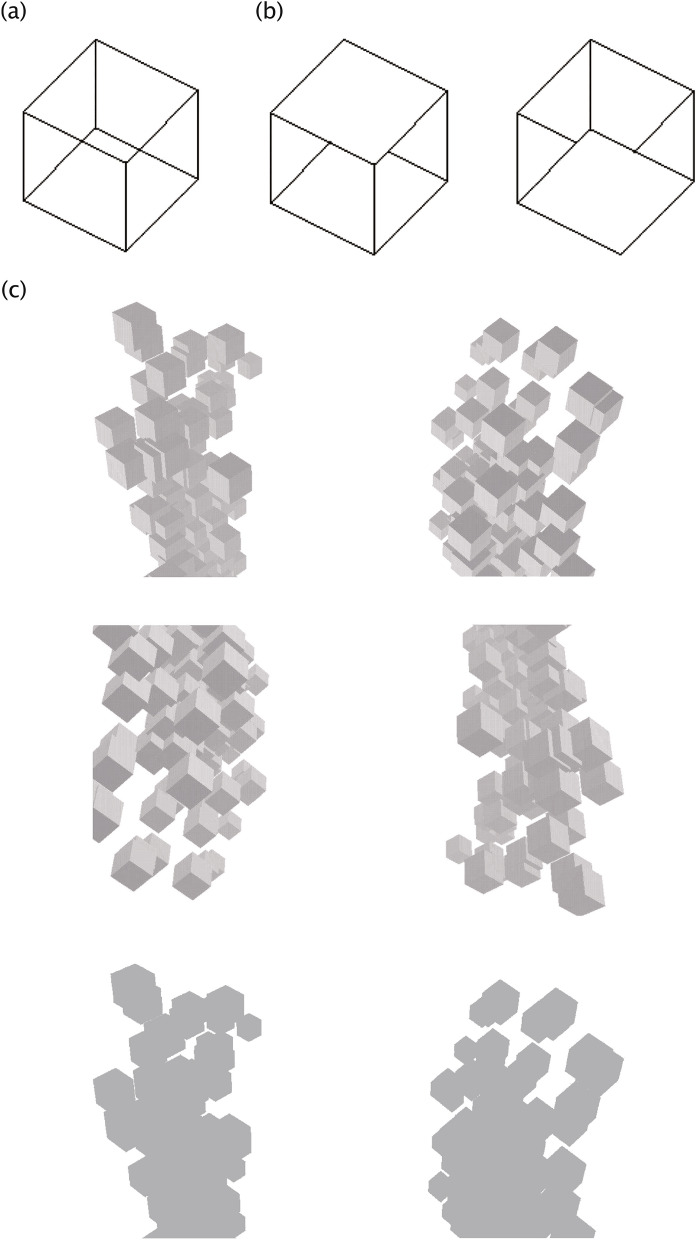
Stimuli used in Experiments 1 and 2. **(a)** The main stimulus: A Necker cube stimulus was used that evoked bistable perception from above and below. **(b)** Viewed from above (VFA) (left) and viewed from below (VFB) (right) cubes. In Experiment 1, they were presented before stimulus **(a)** as a cue. They were created by removing several lines from **(a)** to bias perception to VFA or VFB. These stimuli were the same when inverted, and stimuli properties were the same. **(a,b)** were drawn with white edges in the experiment. **(c)** Examples of background contexts used in Experiment 2. One of the three background contexts (VFA: top, VFB, middle, control; bottom) was presented around the stimulus of **(a)** randomly. The VFA and VFB stimuli are the same when inverted. The control stimulus was filled to have the same luminance as the average luminance of other background stimuli.

We used three kinds of images in Experiment 1, all of which were generated based on a previous study^17^. The first image was a wireframe drawn Necker cube (Fig. 1a). The second and third images were unambiguous cubes, one as VFA (Fig. 1b) and the other as viewed from below (VFB; Fig. 1c). The color of the cube edges was white (R, G, B = 255, 255, 255) on a gray background (R, G, B = 128, 128, 128). These Necker cubes were placed in the virtual reality (VR) space at a distance of 100 Units with a size of 4 × 4 Units (“Unit” is an arbitrary measurement representing length in the Unity environment: 1 Unit is approximately 1 m), and the visual angle was 2.29 × 2.29 degrees. The fixation cross was black (R, G, B = 0, 0, 0) with a 1.15 × 1.15 visual angle. These images were first created with GIMP and adjusted with Unity.

In Experiment 2, we used the Necker cube and three background contexts (Fig. 1a,c). One context simulated the appearance from above (Fig. 1c Top), and the second simulated the appearance from below (Fig. 1c Middle). These two were flipped upside down and had the same brightness. The third background context was a gray-filled cube with no upper or lower cue created by Matlab (Fig. 1c Bottom). The average luminance of these stimuli was 37.61 cd/m^2^. The Necker cubes were placed in the VR space at a distance of 100 Units with a size of 2 × 2 Units, and the visual angle was 1.15 × 1.15. These contents were placed in the VR space at a distance of 100 Units with a size of 12 × 24 Units, and the visual angle was 6.89 × 13.69.

All stimuli were shown on an HMD (HTC VIVE, HTC Corporation, Taiwan) at 2160 × 1200 pixels with a refresh rate of 90 Hz. An HTC VIVE controller (HTC Corporation, Taiwan) was used to obtain participants’ behavioral responses.

### Procedure

First, participants wore an HMD and adjusted the head belt according to the size of their heads. Thereafter, a five-point calibration was performed to determine the positions of both eyes and eye gaze. The interpupillary distance was set to 64.1 mm, which is the average interpupillary distance for Japanese men^33^. Participants were seated in a chair and given a VIVE controller to hold. The experiment was performed in a 3D virtual space, but the stimuli were presented as a planar image. The fixation cross was shown for 1000 ms, and then a cube with either the top or bottom side rendered opaque was presented for 1000 ms as a cue (hereafter called “cue”). Two kinds of images were randomly used as cues: one was the perspective of a cube seen from above and the other a perspective from below.

**Figure 2:**
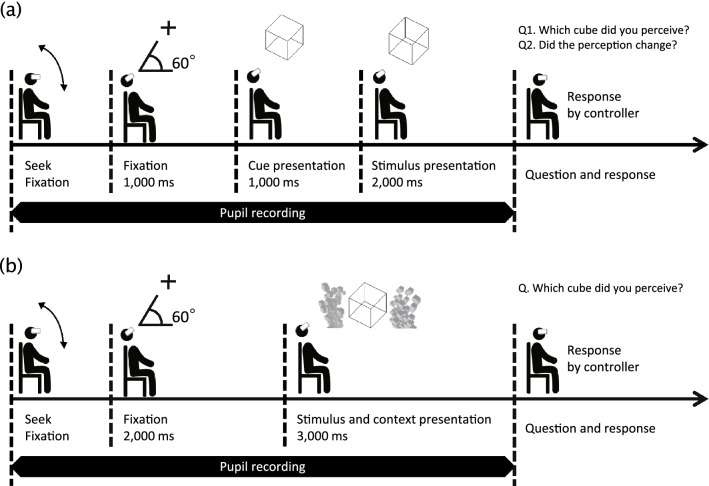
Illustration of the timeline of the experiments. **(a)** Flow of one trial in Experiment 1. Participants shook their heads and looked for a fixed fixation point at one of the five angles. In this example, the trial is for 60°. After gazing at the fixation point for 1000 ms, a cue was presented for 1000 ms, and then the stimulus was presented for 2000 ms. Participants then responded regarding their perception. **(b)** The flow of one trial in Experiment 2. The general flow was similar to Experiment 1, but the fixation point was presented for 2 s, and the stimulus and background context were presented for three seconds concurrently. The question has been simplified to one. Please also see Supplementary File 1.

After the cue, the standard Necker cube was shown (by removing the surface shading while leaving the standard wireframe) for 2000 ms. At its offset, participants reported which percept they saw initially and whether a reversal to the alternative perspective occurred while viewing the empty cube by pressing one of two forced-choice keys. The experiment consisted of two blocks: vertical and horizontal conditions according to the stimulus presentation positions. The stimuli presentation angle was set to 0° with the head horizontal to the ground, and angle conditions were set to −60°, −30°, 0°, 30°, and 60° (vertical and horizontal) in each block. There were 20 total conditions (each cue type × five angle types × vertical and horizontal block), with 16 trials each, for a total of 320 trials. Trials in the block were randomized and divided into four sessions, and sufficient breaks were given between sessions. The order between two blocks was counterbalanced by inverting it for every other participant. Participants were instructed to look at the center of the stimuli as much as possible during each trial. The timeline of one trial in Experiment 1 is shown in Fig. 2a.

In Experiment 2, the background context and the Necker cube stimulus were used to investigate the relationship between the background context and posture. A fixation point was presented for 2000 ms, then a Necker cube with one of the three contexts was shown for 3000 ms, and then participants responded to the appearance of the Necker cube. The stimuli presentation angle was set to 0° with the head horizontal to the ground, and angle conditions were set to −60°, −30°, 0°, 30°, and 60° in each block. There were 15 total conditions (each context type [VFA, VFB, control] × five angle types), with 16 trials each, for a total of 240 trials. The timeline of one trial in Experiment 2 is shown in Fig. 2b.

### Behavioral analysis

From the participants’ key-press responses, we calculated the probability that they perceived the VFA appearance of the cube. A three-way repeated-measures ANOVA was conducted using the average probabilities for each cue (VFA and VFB), each angle (−60, −30, 0, 30, 60), and each direction (vertical and horizontal) as within-subject factors in Experiment 1. A two-way repeated-measures ANOVA was conducted using the average probabilities for each angle (−60, −30, 0, 30, 60) and each context (VFA, VFB, control) as within-subject factors in Experiment 2. Pairwise comparisons for main effects were corrected for multiple comparisons using Shaffer’s modified sequentially rejective Bonferroni (MSRB) method, and the level of statistical significance was set to *p* < 0.05 for all analyses. The Greenhouse–Geisser corrections were performed when the results of Mauchly’s sphericity test were significant. The data were analyzed using Matlab 2018b (MathWorks, Natick, MA, USA) and R (4.0.2) with a tool for ANOVA (anovakun version 4.8.5).

### Pupil recording and analysis

Pupil sizes and eye movements were measured during the task with optional corrective lenses (VIVE Pro Eye with Tobii eye tracking, Tobii, Sweden) at a sampling rate of 120 Hz. Eye movements were monitored from both eyes. For analyses, we used pupil diameters of the left eye. Interpolation was performed when pupil diameter data could not be obtained due to eye blinking using cubic spline interpolation. Pupil recordings were smoothed using a sliding average (83.3 ms time window). Trials with a change in pupil diameter of more than 0.06 mm/ms were assumed to be artifacts and were excluded from the analysis. One participant’s data had over 50% of trials removed and were thus excluded from the analysis in Experiment 1. In the time course analysis, each trial was normalized by subtracting pupil size at stimulus onset from the baseline pupil size. Baseline pupil size was computed as an average of data collected 200 ms prior to the stimulus onset (0 ms). This onset refers to the cue presentation in Experiment 1 and the stimulus presentation in Experiment 2. We calculated the time course of the trial’s average pupil size in all conditions (two cues, five angles, and two directions in Experiment 1). Specifically, the average pupil diameters from 1000  to 3000 ms after the cue presentation were calculated (during the stimulus presentation for two seconds), and a three-way repeated-measures ANOVA was performed to assess the presence of significant differences in pupil diameter with cue (VFA, VFB), angle (−60, −30, 0, 30, 60) and direction (vertical and horizontal) as within-subject factors in Experiment 1. Similarly, in Experiment 2, the average pupil diameter was calculated, and a two-way repeated-measures ANOVA was performed to assess the presence of significant differences in pupil diameter, with angle (−60, −30, 0, 30, and 60) and context (VFA, VFB, and control). Pairwise comparisons for main effects in the ANOVA were corrected for multiple comparisons using Shaffer’s MSRB method, and the level of statistical significance was set to *p* < 0.05 for all analyses.

## Results

### Experiment 1

**Figure 3:**
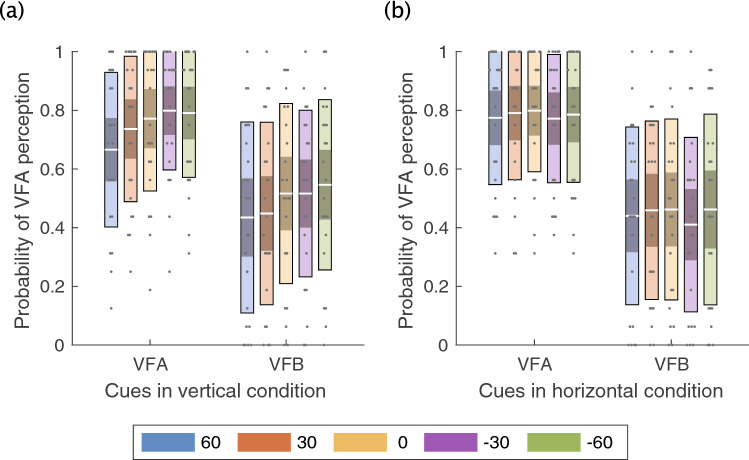
Behavioral results in Experiment 1. **(a)** The average probability of viewed from above (VFA) perception between cue and angle conditions in the vertical condition across all participants. **(b)** The average probability of VFA perception between cue and angle conditions in the horizontal condition across all participants. The white line indicates the mean of participants, the light color indicates 1.96 standard error of the mean (95% confidence interval), and the dark color indicates one standard deviation. Each gray dot indicates the mean of each participant. Each color represents the angle at which the stimulus was presented.

We first analyzed the average probability of VFA perception in each condition (Fig. 3). A three-way ANOVA revealed a significant first-order interaction of average probability of VFA perception between direction and angle (${F}_{(3.29, 72.39)}=4.29, p=0.006,{{\upeta }_{p}}^{2}=0.16$). Subsequent analysis showed that there was a simple main effect for angle in the vertical condition (${F}_{(2.55, 56.04)}=6.29, p=0.002,{{\eta }_{p}}^{2}=0.22$) (Fig. 3a). Importantly, following a multiple comparison for angle in the vertical condition, the probability of VFA perception at −30° and −60° was greater than at 60° ($t\left(22\right)=3.33, p=0.003,{ p}_{adj}=0.03; t\left(22\right)=3.11, p=0.005,{ p}_{adj}=0.03, \, {\rm respectively}$). The ANOVA also revealed a significant main effect of the cue and angle condition (cue: ${F}_{(1, 22)}=10.76, p=0.003,{{\eta }_{p}}^{2}=0.32$; angle: ${F}_{(1, 22)}=5.40, p=0.003,{{\eta }_{p}}^{2}=0.20$). All other conditions and their interactions were nonsignificant.

**Figure 4:**
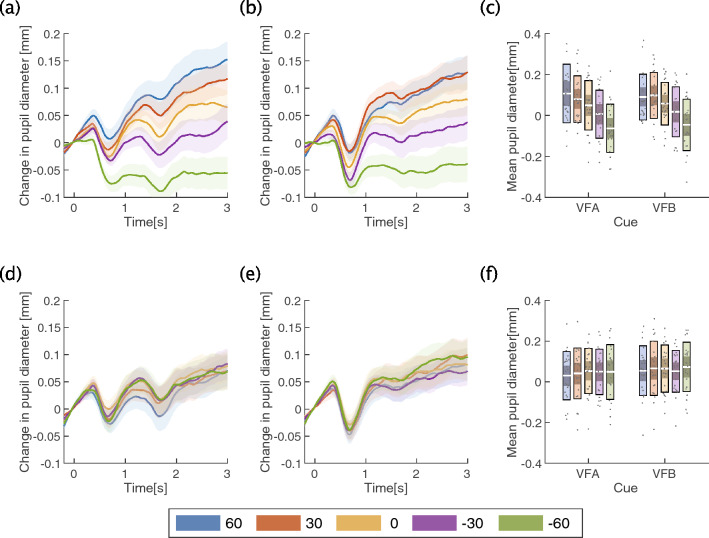
Pupillary results in Experiment 1. **(a)** Time course of average pupil diameter when viewed from above (VFA) was cued in the vertical condition across all participants. **(b)** Time course of average pupil diameter when viewed from below (VFB) was cued in the vertical condition across all participants. **(c)** Average pupil diameter from 1 to 3 s in the vertical condition. **(d)** Time course of average pupil diameter when VFA was cued in the horizontal condition across all participants. **(e)** Time course of average pupil diameter when VFB was cued in the horizontal condition across all participants. **(f)** Average pupil diameter from one to three seconds in the horizontal condition. In **(a,b,d,e)**, the line shows the average pupil diameter, and the shaded color shows the standard error of the mean (SEM). In these figures, the cues were presented in the range from 0 to 1 s, and the ambiguous Necker cube was presented from one to three seconds (the range of baseline was −200 ms to 0 s, which was presented as the fixation point). In (**c,f**), the white line indicates the mean of participants, the light color indicates 1.96 SEM (95% confidence interval), the dark color indicates one standard deviation, and each gray dot indicates the mean of each participant. Each color represents the angle at which the stimulus was presented.

We then analyzed and compared the pupil diameter between the conditions (Fig. 4). The three-way ANOVA revealed a significant first-order interaction of average pupil diameter between direction and angle (${F}_{\left(2.40, 50.36\right)}=20.26, p<0.0001,{{\eta }_{p}}^{2}=0.49$). Subsequent analysis showed that there was a simple main effect for angle in the vertical condition (${F}_{\left(2.39, 50.21\right)}=27.24, p<0.0001,{{\eta }_{p}}^{2}=0.56$) (Fig. 4c). Importantly, following a multiple comparison for angle in the vertical condition, pupil diameter at −60° was smaller than in all other conditions (vs. 60: $t\left(21\right)=6.31, p<0.0001,{ p}_{adj}<0.0001;$ vs. 30: $t\left(21\right)=6.95, p<0.0001,{ p}_{adj}<0.0001;$ vs. 0: $t\left(21\right)=5.89, p<0.0001,{ p}_{adj}<0.0001;$ vs. −30: $t\left(21\right)=5.05, p=0.0001,{ p}_{adj}=0.0003$) (Fig. 4c). Moreover, following a multiple comparison for angle in the vertical condition, pupil diameter at −30° was smaller than at 60°, 30° and 0° ($t\left(21\right)=4.45, p=0.0002,{ p}_{adj}=0.0009; t\left(21\right)=5.23, p<0.0001,{ p}_{adj}=0.0002;$ $t\left(21\right)=3.74, p=0.0012,{ p}_{adj}=0.0049, {\rm respectively}$). The multiple comparison also showed pupil diameter at 0 degrees was smaller than at 60° and 30° ($t\left(21\right)=2.77, p=0.0114,{ p}_{adj}=0.0342;$ $t\left(21\right)=2.60, p=0.0167,{ p}_{adj}=0.0342, {\rm respectively}$). On the other hand, a simple main effect for the direction and angle interaction was not significant in the angle in the horizontal condition (${F}_{\left(2.90, 60.96\right)}=0.78, p=0.504,{{\eta }_{p}}^{2}=0.03$). Because we included a large number of factors, the results were complicated, and only the important results are presented here (see the tables in the supplementary material for all statistical results).

Taken together, our results suggested that the perception of Necker cubes changed depending on neck posture; however, there were two concerns. The first was the dynamics in the pupil diameter of early latency at the time of cueing, which may have reflected the noise of neck movement before stimulus presentation. The second was that the pupillary responses might include the effect of the cueing stimulus itself. To address this problem, in Experiment 2, we aimed to reduce movement noise by extending the head fixation time by two seconds. In addition, we tested whether the same effect could be obtained by changing the background context instead of cueing to confirm it was not a cue-specific effect.

### Experiment 2

**Figure 5:**
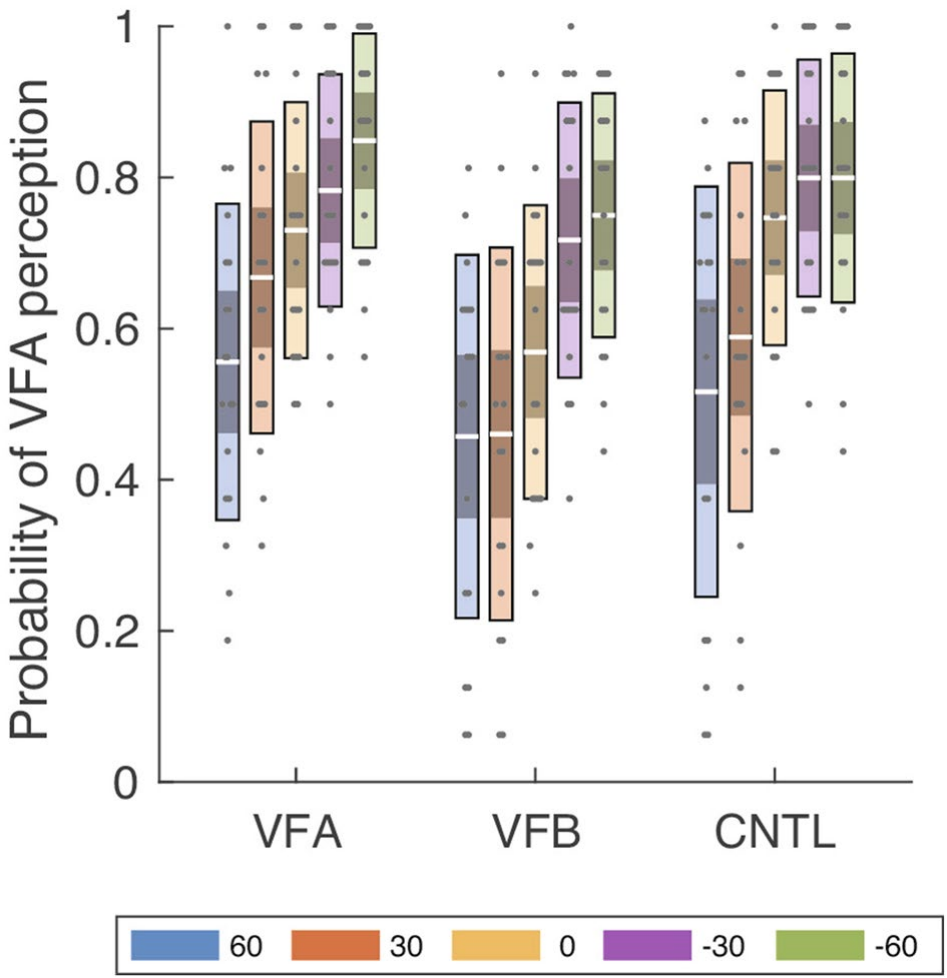
The average probability of viewed from above (VFA) perception between the cue and angle conditions across all participants in Experiment 2. The white line indicates the mean of participants, the light color indicates 1.96 standard error of the mean (95% confidence interval), and the dark color indicates one standard deviation. Each gray dot indicates the mean of each participant. Each color represents the angle at which the stimulus was presented.

Similar to Experiment 1’s analysis, we calculated and analyzed the average probability of VFA perception in each condition (Fig. 5). A two-way ANOVA showed a significant main effect of the probability in the context and angle conditions (context: ${F}_{\left(1.41, 25.35\right)}=13.93, p=0.0003,{{\eta }_{p}}^{2}=0.44$; angle:${F}_{\left(1.44, 25.98\right)}=13.77, p=0.0003,{{\eta }_{p}}^{2}=0.43$). A multiple comparison of context showed that the probability in the VFB context was significantly smaller than in the VFA and control contexts (VFB vs. VFA:$t\left(18\right)=4.02, p=0.0008,{ p}_{adj}=0.0024; \mathrm{VFB vs}.\mathrm{ control}: t\left(18\right)=3.91, p=0.0010,{ p}_{adj}=0.0024$). In addition, a multiple comparison for the angle condition showed that the probability at −60° and −30° was greater than at 0°, 30°, and 60° (−60° vs. 0°: $t\left(18\right)=4.73, p=0.0002,{ p}_{adj}=0.0017;$ −60 vs. 30: $t\left(18\right)=4.53, p=0.0003,{ p}_{adj}=0.0017; 60\mathrm{ vs}. -60: t\left(18\right)=4.43, p=0.0003,{ p}_{adj}=0.0019;$ −30 vs. 0: $t\left(18\right)=4.73, p=0.0002,{ p}_{adj}=0.0017;$ −30 vs. 30: $t\left(18\right)=4.12, p=0.0006,{ p}_{adj}=0.0039;$ −$30 \mathrm{vs}. 60: t\left(18\right)=3.99, p=0.0009,{ p}_{adj}=0.0039$). All other conditions and their interactions were nonsignificant.

**Figure 6:**
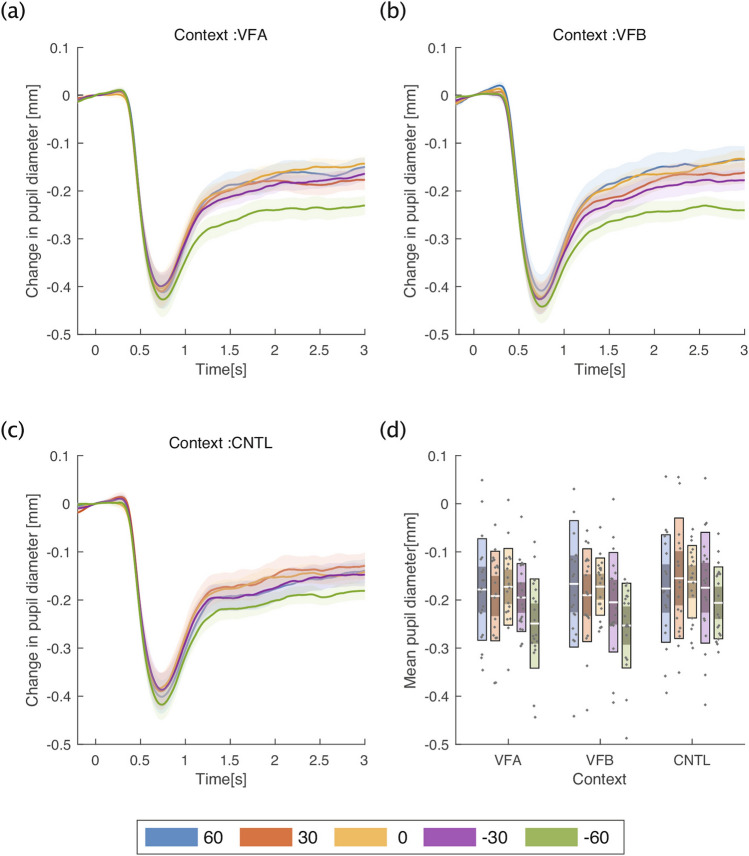
Pupillary results in Experiment 2. (**a**) Time course of average pupil diameter in the viewed from above (VFA) context across all participants. (**b**) Time course of average pupil diameter in the viewed from below (VFB) context across all participants. (**c**) Time course of average pupil diameter with the control context across all participants. (**d**) Average pupil diameter from one to three seconds for each context in the vertical condition. In (**a,b**), the line shows the average pupil diameter, and the shaded color shows the standard error of the mean (SEM). In (**c**), the white line indicates the mean of participants, the light color indicates 1.96 SEM (95% confidence interval), the dark color indicates one standard deviation, and each gray dot indicates the mean of each participant. Each color represents the angle at which the stimulus was presented.

The pupillary data were also analyzed for each condition as in Experiment 1 (Fig. 6). A two-way ANOVA showed a significant main effect of average pupil diameter both in the context and angle conditions (context: ${F}_{\left(1.69, 30.49\right)}=6.10, p=0.0083,{{\eta }_{p}}^{2}=0.25$; angle:${F}_{\left(2.32, 41.73\right)}=8.11, p=0.0006,{{\eta }_{p}}^{2}=0.31$) (Fig. 6c). Interestingly, a multiple comparison for the context condition showed that the average pupil diameter in the control context was significantly greater than in the VFA and VFB contexts, unlike the behavioral results (control vs. VFA $t\left(18\right)=2.55, p=0.0197,{ p}_{adj}=0.0197;$ control vs. VFB $t\left(18\right)=3.71, p=0.0016,{ p}_{adj}=0.0048)$. Additionally, a multiple comparison for the angle condition showed that the average pupil diameter at −60° was significantly smaller than at all other angles (−60 vs. 60 $t\left(18\right)=3.32, p=0.0038,{ p}_{adj}=0.0230;$ −60 vs. 30: $t\left(18\right)=3.62, p=0.0020,{ p}_{adj}=0.0118;-60\mathrm{ vs}. 0: t\left(18\right)=5.67, p<0.0001,{ p}_{adj}=0.0002;-60\mathrm{ vs}.-30: t\left(18\right)=4.82, p=0.0001,{ p}_{adj}=0.0008)$. All other conditions and their interactions were nonsignificant.

## Discussion

### Neck posture modulates the effects of perceptual bias

The purpose of this study was to clarify the relationship between neck posture and visual heuristics and investigate the perception of the appearance of Necker cubes placed in VR space in various neck postures. In Experiment 1, when looking down (at −60° and −30°), the probability of VFA perception of the Necker cube was significantly greater than when looking up (at 60°). The same effect was replicated in Experiment 2 (VFA probability at −60° and −30° was greater than 0°, 30°, and 60° in the angle condition). Essentially, our experiments demonstrated that the effects of perceptual bias differ depending on neck posture even though the stimuli presented on the retina were the same. Interestingly, the degree of neck movement did not affect initial pupil response in the horizontal condition.

In a previous study that investigated perception with the head in an inverted state (i.e., viewing from between the legs), the difference in perception from the normal posture was explained by the change in the proprioceptive sensation of the head in an abnormal state^7,8^. In our experiments, the normal neck angle used in daily life was adopted, but the effect of perceptual bias was changed. Thus, the proprioceptive sensation of the head is more sensitive than previously thought and may affect perception even in normal postures.

In addition, our results can be interpreted using the Bayesian theory of perception^34^. In the present study, the interpretation of the Necker cube was explained by interpretation parameters combined, prior and posterior distribution, over time as a perceptual decision-making model. That is, we consider that neck posture is incorporated in the Bayesian theory of perception as a variable that influences perceptual stability. To illustrate, in daily life, we look down to see the appearance of the VFA cube. Conversely, the VFB cube can also be seen by looking up. Thus, it can be interpreted that such perceptual experience and posture are linked and affect long-term memory related to our perception.

### Pupil diameter size is consistent with perception probability

Interestingly, pupil diameter was significantly smaller in the looking down condition (in the case of the −60° condition compared with other pupil diameters and in the case of the −30° condition compared with the 0°, 30°, and 60° conditions). Contrary to what we expected, the considerable change of pupil diameter in Experiment 1 occurred before stimulus presentation. This early pupillary response is assumed to be due to neck movement rather than visual stimulation considering the latency of the pupil diameter. Another possible factor influencing pupil dilation was the effect of perceptual switching, which we examined in additional analyses, but it did not seem to be related to differences in angle conditions (see Fig. S5 in the supplementary material). We also checked for systematic bias using absolute values in addition to the extent of change in pupil diameter. However, the results did not correspond to differences in angle conditions (see Fig. S6 in the supplementary material). In addition, to confirm whether fixation stability after stimulus presentation was related to pupil diameter, bivariate contour ellipse areas (BCEAs) of eye movements were calculated. The results showed this factor was also unrelated to angle conditions (see “Analysis of BCEAs” in the supplementary material).

Thus, we conclude that the early pupillary response may have been caused by neck movement. In fact, in Experiment 2, as the head fixation before stimulus presentation was extended to 2 s, the variation of initial pupil response was small compared to Experiment 1 in the angle condition. This difference in early pupil diameter might reflect the locus coeruleus (LC)-norepinephrine system, which has an inhibitory effect on the parasympathetic oculomotor complex through the release of norepinephrine from the LC, which is also involved in postural control^27,35,36^. In addition, noradrenergic LC neurons are also involved in the vestibulo-autonomic reflex^37^. Thus, our results indicate that the modulation of LC activity caused by the postural control of the neck changed pupil diameter.

It is important to further consider the causality between pupillary change and perceptual change. Interestingly, previous studies reported that pupil diameter had a direct effect on the feedforward response in the early visual cortex independent of psychological factors^38^. This poses the following questions: is the change in perception directly related to the physical pupil size modulation? Or is change in pupil diameter simply the outcome of cognitive activity reflecting the subject’s perception? These points should be investigated in future studies.

In Experiment 2, except for the early change in pupil diameter that was expected to be caused by postural control, the average pupil diameter in the control context was significantly greater than in both the VFA and VFB contexts. Since the brightness of these background contexts was equal, it can be deduced that light intensity has almost no physical effect on pupil diameter. Background contexts are known to contribute to perceptual stability^17,39^; therefore, the attentional load is considered to be greater with no context than with a context. In addition, increased attentional effort dilates pupil diameter^23,40,41^. Therefore, our results could be interpreted as the attentional load reflected in the pupil diameter.

This study had several limitations that should be considered. First, the change in pupil diameter was unexpected before the stimulus was presented. In Experiment 2, considering that the baseline was almost the same after two seconds of moving the neck, the change subsided within two seconds after the change in neck posture. However, the exact latency and amount of change in pupil diameter due to the change of the neck remain unclear. Thus, further research is needed to investigate the details of this change in pupil diameter. Second, the vertical movement of the neck caused a difference in perception; however, it was unclear whether this was due to the direction of gravity related to the position of the stimuli—top or bottom—or due to the body coordinate system. Consequently, further research is needed on whether not only neck posture but also whole body posture affects perceptual bias.

## Conclusion

The purpose of this study was to clarify the relationship between neck posture and visual heuristics from the aspects of both behavioral response and pupil diameter, an established physiological cognitive index. Our results showed that the probability of the VFA bias perception of the Necker cube was significantly greater when looking down than when looking up. Interestingly, the pupillary results were also consistent with the probability of perception. These results indicate that perception was modulated by neck posture and suggest that neck posture is incorporated into ecological constraints. To our knowledge, this is the first study to link pupil diameter, perceptual heuristics, and posture and to find that postural changes affect perception and that pupil changes intervene to track the perceptual changes. Further, by investigating cognitive processing in various postures and movements in VR space, the relationship between body and perception will become clearer.

## Supplementary Information

Supplementary Legends.

Supplementary Video.

Supplementary Information.
