# Supplementary material for: Backward and forward neck tilt affects perceptual bias when interpreting ambiguous figures
Source: Sci Rep. 2022 May 4;12:7276. doi: 10.1038/s41598-022-10985-4 (PMC9068752; doi:10.1038/s41598-022-10985-4)
Supplement: Supplementary file 1 — Supplementary Legends. [file 41598_2022_10985_MOESM1_ESM.pdf]

Backward and forward neck tilt affects perceptual bias when interpreting ambiguous figures

Fumiaki Sato[1+], Ryoya Shiomoto[1+], Shigeki Nakauchi[1], Tetsuto Minami[1*,]

[1] Department of Computer Science and Engineering, Toyohashi University of Technology, 1-1 Hibarigaoka Tempaku, Toyohashi, Aichi 441-8580, Japan

+ equally contributed author

*Corresponding author

Supplementary Information contains:

- 1 Supplementary File 1 (Movie:MP4)
- 1 PDF file with: - Legend for Supplementary Movie

Legend for Supplementary File 1. Demonstration of the experiments.

The movie is a demonstration of Experiment 1 and Experiment 2. Note that it looks distorted as it is converted from 3D to 2D.
