# Supplementary material for: Backward and forward neck tilt affects perceptual bias when interpreting ambiguous figures
Source: Sci Rep. 2022 May 4;12:7276. doi: 10.1038/s41598-022-10985-4 (PMC9068752; doi:10.1038/s41598-022-10985-4)
Supplement: Supplementary file 3 — Supplementary Information. [file 41598_2022_10985_MOESM3_ESM.docx]

- All statistical results from the pupillary analysis of Experiment 1 (Figure 4)

The tables below present the results of the three-way ANOVA.

Table S1. All ANOVA results from the pupillary analysis of Experiment 1.

Table S2. Simple effects for direction × angle interaction from the pupillary analysis of Experiment 1.

Table S3. Multiple comparisons for angle in the vertical condition from the pupillary analysis of Experiment 1.

Table S4. Multiple comparisons for the angle condition from the pupillary analysis of Experiment 1.

- Pupillary results in Experiment 1 (baseline correction using 0.8–1 seconds)

To confirm the pupillary response excluding the effect of cue, we calculated the baseline before stimulus presentation (i.e., the last 0.2 seconds of the cue, from 0.8 to 1 s of time shown on the x-axes) and corrected pupil diameter (Figure S1). Importantly, this result also showed a significant difference in the vertical condition, as we mention in the paper (see also ANOVA tables S5).

Figure S1. Pupillary results in Experiment 1. (a) Time course of average pupil diameter when viewed from above (VFA) was cued in the vertical condition across all participants. (b) Time course of average pupil diameter when viewed from below (VFB) was cued in the vertical condition across all participants. (c) Average pupil diameter from one to three seconds in the vertical condition. (d) Time course of average pupil diameter when VFA was cued in the horizontal condition across all participants. (e) Time course of average pupil diameter when VFB was cued in the horizontal condition across all participants. (f) Average pupil diameter from one to three seconds in the horizontal condition. In (a), (b), (d), and (e), the line shows the average pupil diameter, and the shaded color shows the standard error of the mean (SEM). In these graphs, the cues were presented from zero to one second, and the ambiguous Necker cube was presented from one to three seconds (the range of the baseline was 800 ms to one second, which was presented as the fixation point). In (c) and (f), the white line indicates the mean of participants, the light color indicates 1.96 SEM (95% confidence interval), the dark color indicates one standard deviation (SD), and each gray dot indicates the mean of each participant. Each color represents the angle at which the stimulus was presented.

Table S5. ANOVA results from the pupillary analysis of Experiment 1(baseline correction using 0.8–1 seconds).

Table S6. Simple effects for direction × cue interaction from the pupillary analysis of Experiment 1(baseline correction using 0.8–1 seconds).

Table S7. Simple effects for direction × angle interaction from the pupillary analysis of Experiment 1(baseline correction using 0.8–1 seconds).

Table S8. Multiple comparisons for angle in the vertical condition from the pupillary analysis of Experiment 1(baseline correction using 0.8–1 seconds).

Table S9. Multiple comparisons for the angle condition from the pupillary analysis of Experiment 1(baseline correction using 0.8–1 seconds).

- Perceptual switching analysis in Experiment 1

We analyzed the average probability of the rate of perceptual switching in each cue condition, as shown in Figure 3 (Figure S2). Statistical analysis results showed significant main effects for the cue ($F_{(1, 22)}=5.01, p=0.036,{\eta_{p}}^{2}=0.19$) and angle conditions ($F_{(3.56, 78.3)}=3.24, p=0.020,{\eta_{p}}^{2}=0.13$). After multiple comparisons of the angle condition in post hoc analysis, the perceptual switching rate was significantly higher at -60 degrees than at 60 degrees ($t\left( 23 \right)=3.12, p=0.0050,p_{adj}=0.0497$).

Figure S2. Behavioral results of perceptual switching in Experiment 1. (a) The average probability of occurred perceptual switching between cue and angle conditions in the vertical condition across all participants. (b) The average probability of occurred perceptual switching between cue and angle conditions in the horizontal condition across all participants. The white line indicates the mean of participants, the light color indicates 1.96 SEM (95% confidence interval), and the dark color indicates one SD. Each gray dot indicates the mean of each participant. Each color represents the angle at which the stimulus was presented.

However, this analysis did not reveal how many perceptual switches occurred from which perceptual state (VFA or VFB). Therefore, we calculated the rate of the presence or absence of perceptual switches for each perceptual context (VFA and VFB). The figure S3 indicates that the number of perceptual switches among subjects varied.

Figure S3. Behavioral analysis according to perceptual state and perceptual switching. The white line indicates the mean of participants, the light color indicates 1.96 SEM (95% confidence interval), and the dark color indicates one SD. Each gray dot indicates the mean of each participant. Each color represents the angle at which the stimulus was presented.

Next, we conducted pupillary analysis based on the presence or absence of perceptual switches. However, we could not analyze the pupil diameter for each participant (Data were completely lost for some participants. Considering the probability is approximately 0.2 in many conditions in Figure S3, it is appropriate to assume some participants had no perceptual switches.). Therefore, although statistical processing was not possible, we averaged the trials of all participants and illustrated the perceptual state (VFA, VFB) × presence of perceptual alternation (yes, no) in a 2 × 2 diagram.

Figure S4. Pupillary analysis according to perceptual state and perceptual switching in the vertical condition. Time course of average pupil diameter according to perceptual state and perceptual switching in the vertical condition across all trials. The line shows the average pupil diameter, and the shaded color shows the SEM. In these graphs, the cues were presented from zero to one second, and the ambiguous Necker cube was presented from one to three seconds (the range of the baseline was -200 ms to zero seconds, which was presented as the fixation point).

Figure S5. Analysis according to perceptual state and perceptual switching in the horizontal condition. Time course of average pupil diameter according to perceptual state and perceptual switching in the horizontal condition across all trials. The line shows the average pupil diameter, and the shaded color shows the SEM. In these graphs, the cues were presented from zero to one second, and the ambiguous Necker cube was presented from one to three seconds (the range of the baseline was -200 ms to zero seconds, which was presented as the fixation point).

In Figure S5, the presence or absence of perceptual switching did not seem to have a significant effect on pupil diameter. Thus, it seems pupil diameter depends on the vertical neck angle rather than perceptual state.

After completing the analysis of perceptual switching, no new findings were determined, so we did not include this information in the main article.

- Analysis of absolute pupil size

To confirm whether the baseline of pupil diameter which reflects some systematic bias existed from neck posture, we analyzed the dynamics of absolute pupil diameter (not baseline-corrected) (Figure S6). For Figure S6 (a) and (b), we performed repeated ANOVAs to confirm whether there was a significant difference between the 0.8 and 1 second baselines. The ANOVA revealed a significant difference in the angle condition ($F_{\left( 1.85, 38.75 \right)}=3.38, p=0.048 ,{\eta_{p}}^{2}=0.14$). In the post hoc analysis, multiple comparisons of the angle condition showed pupil diameter at -30 degrees was smaller than at -60 degrees ($t\left( 21 \right)=3.38, p=0.0029,p_{adj}=0.0285$). All other conditions and their interactions were nonsignificant. There was a significant difference between -60 and 60 degrees. This difference cannot explain all the differences in the angle conditions in the analysis so far.

Figure S6. Absolute value pupillary analysis results in Experiment 1. (a) Time course of average pupil diameter when VFA was cued in the vertical condition across all participants without baseline correction. (b) Time course of average pupil diameter when VFB was cued in the vertical condition across all participants without baseline correction. (c) Average pupil diameter from 0.8 to one second for each condition in the vertical condition. In (a) and (b), the line shows the average pupil diameter, and the shaded color shows the SEM. In the graphs, the fixation points were presented from -1 to zero seconds, the cues were presented from zero to one second, and the ambiguous Necker cube was presented from one to three seconds. In (c), the white line indicates the mean of participants, the light color indicates 1.96 SEM (95% confidence interval), the dark color indicates one SD, and each gray dot indicates each participant’s mean. Each color represents the angle at which the stimulus was presented. Note that the data of the first 83 ms were removed due to the smoothing bin.

- Analysis of bivariate contour ellipse areas (BCEAs)

We calculated BCEAs for when the target stimuli was viewed in each condition in both Experiments 1 and 2. In summary, there were no significant differences in BCEAs between neck angles and conditions in either experiment. Therefore, it is unlikely that gaze stability influenced our study results. The statistical results are as follows.

To assess the effect of gaze position, we used the BCEA method (Crossland & Rubin, 2002), which is common in eye tracking:

$\mathrm{BCEA}=2k\pi\sigma_{X}\sigma_{Y}\sqrt{1-\rho^{2}}$,

where $k$ is $k=-\text{log}(1-P)$, *P* is set to 0.68 (one SD), $\sigma_{X}$ and $\sigma_{Y}$ are the SDs of the measurement locations along the x and y axes, and ρ is the Pearson product-moment correlation coefficient between x and y (Crossland & Rubin, 2002; Niehorster et al., 2017). The coordinates of the measurement location were calculated by projecting the participant’s gaze vector onto a plane perpendicular to the vector from the participant to the target, which was placed at the target location.

As for Experiment 1, we conducted a two-factor repeated measures ANOVA on the BCEAs in each direction: vertical and horizontal. The results showed that there was no main effect either for head angle ($F(1.19,25.06)=1.84$, $MSE=514,182,207,301.11$, $p=.187$, $\hat{\eta}_{p}^{2}=.081$) or cue conditions ($F(1,21)=1.86$, $MSE=90,543,794,115.63$, $p=.188$, $\hat{\eta}_{p}^{2}=.081$), no significant interaction ($F(1.24,26.10)=1.09$, $MSE=205,344,358,086.45$, $p=.322$, $\hat{\eta}_{p}^{2}=.049$) in the vertical condition, no main effect either for head angle ($F(1.56,32.75)=2.75$, $MSE=216,688,144,891.68$, $p=.090$, $\hat{\eta}_{p}^{2}=.116$) or cue conditions ($F(1,21)=0.00$, $MSE=47,203,926,799.83$, $p=.973$, $\hat{\eta}_{p}^{2}=.000$), and no significant interaction ($F(1.41,29.52)=1.71$, $MSE=331,829,608,839.25$, $p=.203$, $\hat{\eta}_{p}^{2}=.075$) in the horizontal condition. The results for Experiment 2 showed that there was no main effect either for head angle ($F(2.47,44.45)=2.08$, $MSE=413,989,818,443.90$, $p=.127$, $\hat{\eta}_{p}^{2}=.104$) or cue conditions ($F(1.51,27.16)=0.49$, $MSE=315,129,725,347.42$, $p=.565$, $\hat{\eta}_{p}^{2}=.027$) and no significant interaction ($F(1.24,26.10)=1.09$, $MSE=205,344,358,086.45$, $p=.322$, $\hat{\eta}_{p}^{2}=.049$).

The BCEAs for each participant in each condition are presented in the next section.

Crossland, M. D. & Rubin, D. and Gary. S. The Use of an Infrared Eyetracker to Measure Fixation Stability. Optometry Vision Sci 79, 735–739 (2002).

Niehorster, D. C., Li, L. & Lappe, M. The Accuracy and Precision of Position and Orientation Tracking in the HTC Vive Virtual Reality System for Scientific Research. i-Perception 8, 204166951770820–23 (2017).

- BCEAs for each participant in each condition

Vertical condition with VFA cue in Experiment 1

Vertical condition with VFB cue in Experiment 1

Horizontal condition with VFA cue in Experiment 1

Horizontal condition with VFB cue in Experiment 1

VFA context in Experiment 2

VFB context in Experiment 2

Control context in Experiment 2
